# SEAI: Social Emotional Artificial Intelligence Based on Damasio’s Theory of Mind

**DOI:** 10.3389/frobt.2018.00006

**Published:** 2018-02-07

**Authors:** Lorenzo Cominelli, Daniele Mazzei, Danilo Emilio De Rossi

**Affiliations:** ^1^E. Piaggio Research Center, Information Engineering Department, University of Pisa, Pisa, Italy; ^2^Computer Science Department, University of Pisa, Pisa, Italy

**Keywords:** cognitive systems, artificial intelligence, artificial consciousness, social robotics, humanoids, somatic markers, rules engine, expert systems

## Abstract

A socially intelligent robot must be capable to extract meaningful information in real time from the social environment and react accordingly with coherent human-like behavior. Moreover, it should be able to internalize this information, to reason on it at a higher level, build its own opinions independently, and then automatically bias the decision-making according to its unique experience. In the last decades, neuroscience research highlighted the link between the evolution of such complex behavior and the evolution of a certain level of consciousness, which cannot leave out of a body that feels emotions as discriminants and prompters. In order to develop cognitive systems for social robotics with greater human-likeliness, we used an “understanding by building” approach to model and implement a well-known theory of mind in the form of an artificial intelligence, and we tested it on a sophisticated robotic platform. The name of the presented system is SEAI (Social Emotional Artificial Intelligence), a cognitive system specifically conceived for social and emotional robots. It is designed as a bio-inspired, highly modular, hybrid system with emotion modeling and high-level reasoning capabilities. It follows the deliberative/reactive paradigm where a knowledge-based expert system is aimed at dealing with the high-level symbolic reasoning, while a more conventional reactive paradigm is deputed to the low-level processing and control. The SEAI system is also enriched by a model that simulates the Damasio’s theory of consciousness and the theory of Somatic Markers. After a review of similar bio-inspired cognitive systems, we present the scientific foundations and their computational formalization at the basis of the SEAI framework. Then, a deeper technical description of the architecture is disclosed underlining the numerous parallelisms with the human cognitive system. Finally, the influence of artificial emotions and feelings, and their link with the robot’s beliefs and decisions have been tested in a physical humanoid involved in Human–Robot Interaction (HRI).

## 1 Introduction

Everyone has a rough idea of what is meant by consciousness, but it is better to avoid a precise definition of consciousness because of the dangers of premature definition. Until the problem is understood much better, any attempt at a formal definition is likely to be either misleading or overly restrictive, or both. (Crick and Clark, 1994)

After many years from these words, consciousness is still a thorny and mysterious subject. In human history, almost every philosopher, religious figure, psychologist, and scientist tried to explain its phenomenology. From Plato and Aristotle to Popper and Searle passing through Descartes and Kant, everyone has attempted to pinpoint the “seat of consciousness.” Today, this is considered as a process in the body–brain complex, from which consciousness arises and takes shape in terms of attitudes, beliefs, desires, and behaviors. If despite the huge advances in computer science, neurophysiology, and brain imaging, we do not have yet a clear vision about this topic, it is because scientific approaches are very recent. For a long time, consciousness has been perceived as something that is not tangible, not measurable, and therefore impossible to afford by means of scientific methods. Fortunately, nowadays, it is well-known that this assumption depended on a rigid distinction between mind and body, highly affected by cultural and religious convictions; merely, an anachronistic and occidental belief, inherited by the Cartesian division between *res cogitans*, a thinking substance which does not occupy physical space, and *res extensa*, our material body. This theory is no further pursued because of the numerous neuroscientists who demonstrated the strict dependency between our body, emotions, feelings, thoughts, and decisions. In particular, the neuroscientist Antonio Damasio demonstrated how strongly emotions and body are interconnected (Damasio, 1994). His theories were supported by studies conducted on brain-injured patients, thanks to which he disclosed how emotions and feelings emerge through the perception of our body, and how this process is fundamental for the arise of our consciousness (Damasio, 2000).

Another fundamental author, who made an important contribution to the understanding of consciousness, is the philosopher and cognitive scientist Daniel Dennett, with his seminal works “Consciousness explained” (Dennett, 1991) and “Kinds of minds: Toward an understanding of consciousness” (Dennett, 1996). In the former, he denied the existence of a single central place deputed to consciousness (the *Cartesian theater*), describing the brain as a “bundle of semi-independent agencies.” In the latter, he led the reader through a fascinating journey in the evolution of living beings to delineate the development of an intelligent conscious mind. He identified this phenomenon with the emergence of capabilities and means that turned out to be advantageous for the interaction between their possessor and the specific environment in which he lives. Therefore, consciousness is explained as the emergence of a set of inner mental representations, which results in the form of intentionality (previously discussed in Dennett (1989)). Clearly, an agent cannot develop any form of intentionality, beliefs, desires, and hence any kind of consciousness, without an autonomous mechanism, which lets him discriminate the entities that share the same environment.

Our purpose is to use an “understanding by building” approach (Webb, 2001) and to treasure all these theories applying them in the field of Social Robotics. In particular, we believe that the Damasio’s three-layered theory of consciousness (Damasio, 2000) is applicable as a cognitive model for artificial intelligence (AI) and that the mechanism of somatic markers (Damasio, 1994) is an adequate mechanism for making an artificial agent able to autonomously interpret the entities of its social environment. When followed as design specifications, these can be the key elements to endow a social robot with the possibility to develop more complex and human-like behavior. Such a novel control architecture, highly human-inspired, would be the beginning of a new social robotics control paradigm.

## 2 Cognitive Systems in Social Robotics

There are different definitions of Social Robot (Dautenhahn and Billard, 1999; Bartneck and Forlizzi, 2004; Breazeal, 2004) but they share fundamental characteristics: all these researchers agree that social robots may have different shapes or functions, but they always have to be able to recognize the presence of humans, engage them in a social interaction, express their own synthetic emotional state, and interpret that of its interlocutors. At the same time, they must be able to communicate in a natural human-like way, which should include also non-verbal language, such as communication by gestures, postures, facial expressions, or any other intuitive way. This definition is still true, but after a few years can be not sufficient anymore. Indeed, in the last decade, there has been a massive increase in the diffusion of social robots, and there have been great advances in the fields in which these robots can be involved. Some of these sectors are personal assistance and support in the house of elderly people (Pineau et al., 2003; Broekens et al., 2009; Sharkey and Sharkey, 2012), robot therapy in the hospitals, e.g., in the treatment of ASD disorder (Werry et al., 2001; Pioggia et al., 2005; Scassellati et al., 2012) and depression (Wada et al., 2005; Alemi et al., 2014), contexts of public service (Chung et al., 2007), and even education (Saerbeck et al., 2010; Causo et al., 2016; Vouloutsi et al., 2016). It is evident that their role is moving further and further away from the traditional role of servants, for assuming more the role of companions in a peer relationship. This leads to the need for enhancing some of their requirements, such as empathic behavior, expressiveness, and believability. According to the classification made by Fong et al., it is possible to distribute social robots in a graduated scale that goes from the minimum level of *socially evocative*, robots that rely on the human tendency to anthropomorphize and capitalize on feelings evoked when humans nurture, care, or feel involved with their “creation,” to the highest that is *socially intelligent*, robots that show aspects of human-like social intelligence, based on deep models of human cognition and social competence (Fong et al., 2003). The state-of-the-art of this kind of robots shows great results of social robotics in this direction, but, if we focus on the cognitive system controlling a specific robot, it is always characterized by a specific feature that has been highly developed to the detriment of other functionalities.

Reporting some examples of cognitive systems for social robotics, a well-known case is the one of the cartoon-like robot Kismet (Breazeal and Scassellati, 1999). The underlying architecture of this robot was designed on the base of behavioral models and mechanisms of living creatures, and it is referred by Cynthia Breazeal as “the robot’s synthetic nervous system” (SNS). This modular framework was structured to provide Kismet with the ability to express lifelike qualities, perceive human social behaviors, and allow the robot to be socially situated with people. Nonetheless, the system was intrinsically designed to model the social interaction between an infant and its caregiver, that resulted in a very sophisticated realism, believability, and expressiveness of the robot, but it did not allow the agent to develop specific behaviors toward different interlocutors neither to reason about their emotional state (Breazeal, 2003, 2004). This work was extended on Leonardo, another robot, whose cognitive system was focused on the functionalities of “perspective-taking” and “mind-reading” (Berlin et al., 2006). An infant-like humanoid that can be definitely considered an emotional social robot is iCub (Metta et al., 2010). It is used as an open-systems platform for research in neuroscience and cognitive development but its biologically inspired cognitive system is more oriented on learning and evolution of some fundamental human movement capabilities, such as object tracking and grasping, or learning by demonstration (Vernon et al., 2007).

In many cases, we found that different approaches correspond to a different level of complexity. For example, a strategy to improve the quality of a social interaction, and increase the empathy of the interlocutors, is to move away from complex cognitive architectures and rely more on the effects of a good affordance, as in the case of Paro (Kidd et al., 2006). The opposite direction has been taken by other researchers, who developed ambitious systems that are highly biomimetic. These research groups are trying to reproduce the function of brain areas and neural pathways for mimicking human cognitive capabilities, as in the case of the Distributed Adaptive Control (DAC) (Verschure, 2012), which has been used in applications with iCub, Zeno (Vouloutsi et al., 2016), and Nao (Fernando et al., 2014).

On the side of artificial consciousness, there is a recent review of cognitive systems inspired by how consciousness arise in humans made by Chella and Manzotti (2013) and another even more recent publication written by Dehaene et al. (2017). We strongly agree with the first authors when saying that consciousness could be the missing step in the ladder from current artificial agents to human-like agents. In the second work, Dehaene et al. suggest that the word “consciousness” conflates two different types of information processing computations in the brain: the selection of information for global broadcasting (C1), and the self-monitoring of those computations (C2). They argue that, despite their recent success, current machines are still mostly implementing computations that reflect unconscious processing (C0) in the human brain. We share also this latter analysis. Indeed, all the cognitive architectures that we investigated are extremely advanced works, and each of these systems, or machines, fully satisfies the purpose for which has been conceived. Nonetheless, in none of these instances, we have found a real creation of personal preferences acquired and processed through the body and emotions of the agent, which is considered the base for the foundation of a potential artificial consciousness.

We identify the best explanation of this process in the Damasio’s theory of mind, and we claim that, as yet, the best formalization of this theory is not implemented in any robotic system, but still remains the formalization done by Bosse et al. (2008), which will be introduced in the following section. On the basis of this observation, we decided to design from scratch a novel cognitive architecture for social robotics, which is intended to be the implementation of the Bosse computational model, in order to stay as close to the Damasio’s theory of mind as possible. Then, we will test the resulting system to assess the emergence of some form of artificial consciousness and its repercussions on the social behavior and beliefs of an artificial agent.

## 3 Damasio’s Theory and Its Computational Model

In this section, we will cite several parts from Damasio’s books (Damasio, 1994, 2000), especially the same parts on which Bosse et al. (2008) focused their attention and took inspiration for their formalization. The theory of mind of Antonio Damasio, as well as the way he described the emergence of consciousness, can be seen as the construction of a building. This construction starts from the emotions, passing through feelings, to arrive to what he calls “feelings of feelings.” These are the structural instruments to create the three different levels of consciousness, i.e., respectively: the *proto-self*, the *core consciousness*, and the *extended consciousness*. These three floors share the same building: the body. This latter must be considered not as the theater in which this process takes place, rather, as a necessary means for the generation of consciousness.

According to the general analysis made by Bosse et al. (2008), Damasio described an *emotion (or internal emotional state) as a (unconscious) neural reaction to a certain stimulus, realized by a complex ensemble of neural activations in the brain*. As the neural activations involved often are preparations for (body) actions, as a consequence of an internal emotional state, the body will be modified into an externally observable emotional state. Next, a *feeling* is described as the (still unconscious) sensing of this body state. Finally, *core consciousness* or *feeling a feeling* is what emerges when the organism detects that its representation of its own body state (the proto-self) has been changed by the occurrence of the stimulus: it becomes (consciously) aware of the feeling.

In Damasio (2000), Damasio described this course of events along five steps:

1. *Engagement of the organism by an inducer of emotion, for instance, a particular object processed visually, resulting in visual representations of the object*.
2. *Signals consequent to the processing of the image of the object activate neural sites that are preset to respond to the particular class of inducer to which the object belongs (emotion-induction sites)*.
3. *The emotion-induction sites trigger a number of responses toward the body and toward other brain sites, and unleash the full range of body and brain responses that constitute emotion*.
4. *First-order neural maps in both subcortical and cortical regions represent changes in body state. Feelings emerge*.
5. *The pattern of neural activity at the emotion-induction sites is mapped in second-order neural structures. The proto-self is altered because of these events. The changes in proto-self are also mapped in second-order neural structures. An account of the foregoing events, depicting a relationship between the “emotion object” (the activity at the emotion-induction sites) and the proto-self is, thus, organized in second-order structures*.

Bosse, Junker, and Treur conceived a model, based on these Damasio’s notions to simulate the dynamics of the basic mechanisms taking place in the mind and body of an agent. These dynamics are described as an evolution of *states* over time. States are intended as neurological states formed by neural processes. They used the following forms of abstraction:

- neural states or activation patterns are modeled as single *state properties*;
- large multi-dimensional vectors of such (distributed) state properties are composed to one single composite state property, when appropriate; e.g., (p1, p2, …) to p and (S1, S2, …) to S.

To describe the dynamics of these processes, Bosse et al. used an explicit reference to time: *dynamic properties* can be formulated relating a state at one point in time to a state at another point in time. They reported the following example “*at any point in time t_1_, if the agent observes rain at t_1_, then there exists a point in time t_2_ after t_1_ such that at point t_2_ the agent has internal state property s*” (Bosse et al., 2008). Where *s*, in the example, is viewed as a *sensory representation* of the rain. These dynamic properties are expressed in a temporal language, i.e., the Temporal Trace Language (TTL) (Jonker et al., 2003), in which explicit references are made to time points and traces. A *trace* over a state is a time-indexed sequence of states. For performing experiments, they exploited a simpler temporal language called Language and Environment for Analysis of Dynamics by SimulaTiOn (LEADSTO) (Bosse et al., 2005). In this way, they can specify simulation models in a declarative manner. A basic notation of LEADSTO is *α* → *e, f, g, h, β*, meaning: “if state property *α* hold for a time interval with duration *g*, then after some delay (between *e* and *f*) state property *β* will hold for a time interval of length *h*” (Herlea et al., 1999).

**Figure 1:**
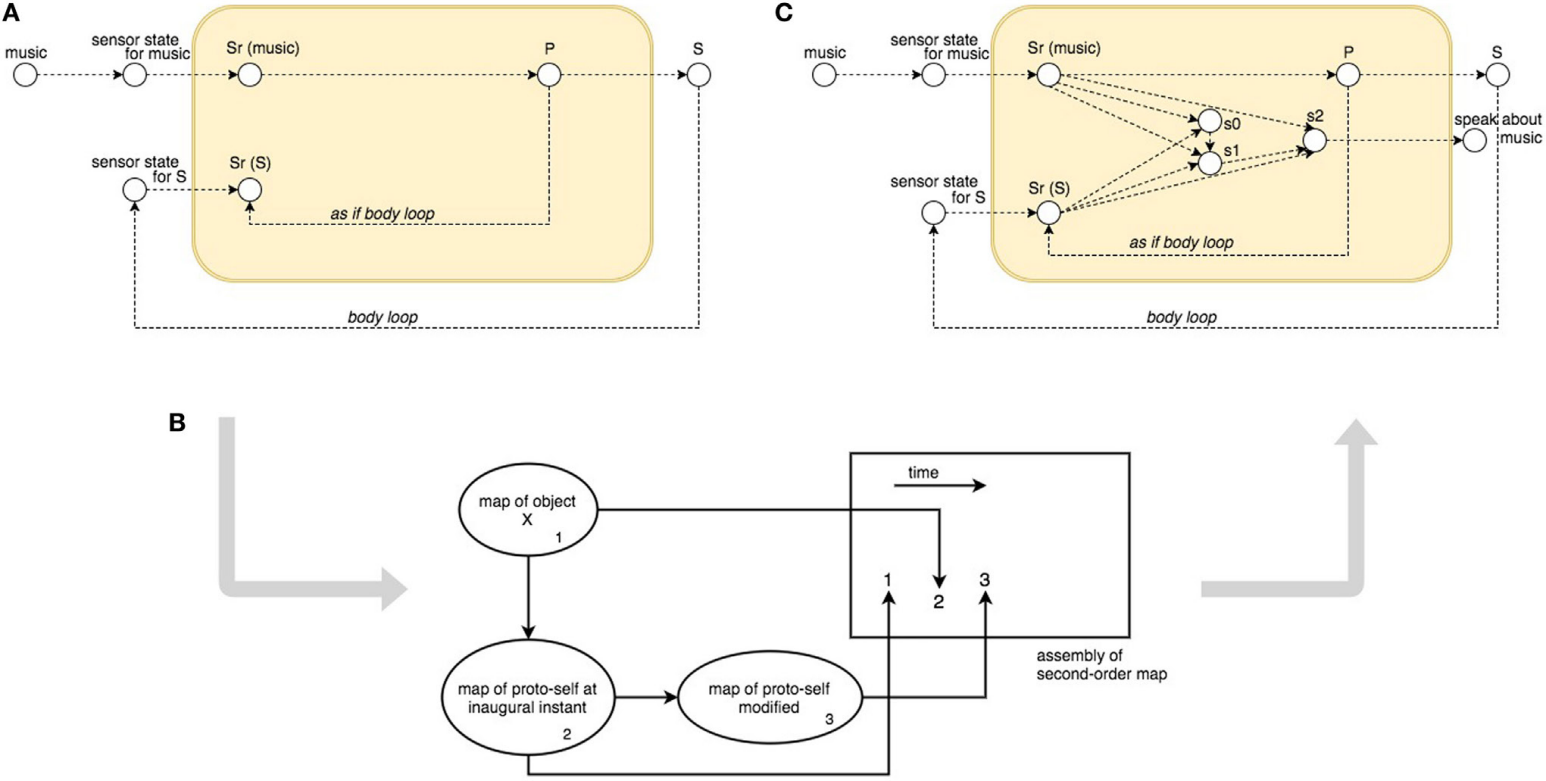
The Bosse et al. computational model: **(A)** *Body loop* and *As If Body Loop* in the generation of feeling; **(B)** Damasio’s picture for assembly of a secondary-order map; **(C)** overview of the overall simulation model.

Relying on this descriptive model, they presented a case in which an agent hears some music, which leads to an emotional state that implies physical responses. The process is described by executable Local dynamic Properties (**LP**) in LEADSTO notation, taking into account internal state property sr(music) for activated sensory representation of hearing the music, and a vector p = (p1, p2, …) of preparation state properties for the activation of the physical responses, defined as the multidimensional composite state property S = (S1, S2, …). A schema of this process is shown in Figure 1A, where the corresponding **LP**s are:

**LP0** music → sensor_state(music)

**LP1** sensor_state(music) → sr(music)

**LP2** sr(music) → p

**LP3** p → S

What is described until **LP3** is the emotional unconscious reaction to a stimulus (or a combination of stimuli), which becomes apparent in the form of bodily changes. According to Damasio (2000), there is still no sense of self nor feelings at this stage, because “*the sense of self has a pre-conscious biological precedent, the proto-self, and (*…*) the earliest and simplest manifestations of self emerge when the mechanism which generates core consciousness operates on that non-conscious precursor*.”

Here is the point in which body and, particularly, changes in the bodily state perceived as emotions assume their fundamental role for the emergence of feelings, which is described as follows: “*as for the internal state of the organism in which the emotion is taking place, it has available both the emotion as neural object (the activation pattern at the induction sites) and the sensing of the consequences of the activation, a feeling, provided the resulting collection of neural patterns becomes images in mind*” (Damasio, 2000).

Therefore, a feeling emerges when the collection of neural patterns contributing to the emotion lead to mental images. In other words, the organism senses the consequences of the emotional state. This result can be achieved by means of two mechanisms described by Damasio as *via the body loop* and *via the as if body loop*. Bosse, abstracting from the detailed steps made of biological states, summarized these two mechanisms as follows:

**Via the *body loop***: the internal emotional state leads to a changed state of the body, which subsequently, after sensing, is represented in somatosensory structures of the central nervous system;

**Via the *as if body loop***: the state of the body is not changed. Instead, on the basis of the internal emotional state, a changed representation of the body is created directly in the sensory body maps. Consequently, the organism experiences the same feeling as via the body loop: it is “as if” the body had really been changed but it was not.

This part is formalized including in the model a number of internal state properties for sensory representation of body state properties (sr(S)) that are changed due to responses to the stimulus. Together, these sensory representations constitute the feeling induced by the stimulus. As shown in Figure 1, sr(S) can be reached in two ways, in LEADSTO notation:

**LP4** S → sensor_state(S)

**LP5** sensor_state(S) → sr(S)

or

**LP6** p → sr(S)

where local dynamic properties **LP4** and **LP5** represent the *body loop*, while **LP6** stands for the *as if body loop*.

Finally, Bosse et al. (2008) faced the consciousness problem of “feeling a feeling.” Damasio described the origin of consciousness with these words: “*Core consciousness occurs when the brain’s representation devices generate an imaged, nonverbal account of how the organism’s own state is affected by the organism’s processing of an object, and when this process enhances the image of the causative object, thus placing it in a spatial and temporal context (p. 169) (*…*) beyond the many neural structures in which the causative object and the proto-self changes are separately represented, there is at least one other structure which re-represents both proto-self and object in their temporal relationship and thus represents what is actually happening to the organism: proto-self at the inaugural instant; object coming into sensory representation; changing of inaugural proto-self into proto-self modified by object (p. 177)*” (Damasio, 2000).

Bosse formalized this final part of the process as transitions between the following moments: **(1)** the proto-self at the inaugural instant; **(2)** an object come into sensory representation; **(3)** the proto-self has become modified by the object (see Figure 1B). Time is once again the key, and Bosse modeled these steps as a temporal sequence, a *trace*: “(…) in the trace considered subsequently the following events take place: no sensory representations for music and S occur, the music is sensed, the sensory representation sr(music) is generated, the preparation representation p for S is generated, S occurs, S is sensed, the sensory representation sr(S) is generated.” To model this process, Bosse et al. (2008) introduced three further internal state properties called: s0 for encoding the initial situation, and s1 and s2 for encoding the situation after two relevant changes. The extended model is depicted in Figure 1C, formalized by the following LEADSTO notation:

**LP7** not sr(music) & not sr(S) → s0

**LP8** sr(music) & not sr(S) & s0 → s1

**LP9** sr(music) & sr(S) & s1 → s2

**LP10** s2 → speak_about(music)

The final state speak_about(music) is an action made by a conscious agent, who is aware of a feeling, emerged as a change in its body, associated with the specific object that invoked that change. For giving a practical example, thanks to the described process, a person after feeling shivers on his back due to the listening of a song, can make a statement such as the following: *“I love this song,”* where an association has been consciously created between a specific agent (*“I”*), a specific feeling (*“love”*), and a specific evocative object (*“this song”*).

Until this stage of the model, although Bosse states his intention to use a temporal approach, time has not been used. Indeed, the time parameters of LEADSTO (i.e., *e, f, g, h*) are not yet mentioned in the model, which, so far, has a more logical/causal approach. Then, time constraints are reintroduced to allow a simulation of the model. This choice was necessary to allow their software environment to generate traces in the time dimension and, thus, simulate reactions of the model to a controlled sequence of events. They successfully run an experiment in which they simulate both the body loop and the as if body loop. Finally, they deepened the Damasio’s concept of “representational content” formalizing in TTL the formation of first-order representations, which refer to external states of world and body, and second-order representations, which refer to internal states (other first-order representations) of the proto-self.

We consider the model proposed by Bosse as the most coherent formalization of Damasio’s theory of mind available in the literature. The proof is that we took the mentioned notions as precise instructions for the design of our framework, and numerous references to the model will be made in the next sections. Nonetheless, this model is a purely computational model. It works very well until it is limited to the domain of information processing. When we move to the design of cognitive systems for agents that have to interact in a real environment, new challenging needs and different requirements come out. The real world changes suddenly and unexpectedly, so real-time systems that are involved in real environments must be flexible and always ready to face conflict situations that require solutions. In some cases, the solution has to be quick and responsive. In some other cases, it is required a higher level of reasoning, which can be more abstract, not time-critical, as well as important. In this context, a temporal approach with time constraints is not adequate.

## 4 The SEAI Framework

(…) having a mind means that an organism forms neural representations which can become images, be manipulated in a process called thought, and eventually influence behavior by helping predict the future, plan accordingly, and choose the next action. (Damasio, 1994)

The mind is described as a process in which **inputs** from sensors are converted into **knowledge structures** that allow **reasoning**. These inputs can determine immediate **reactions**, while the results of the reasoning process are internal or external **actions** that together with the *newly generated knowledge* drive feelings, emotions, and behaviors of human beings.

Humans perceive the world and their internal state through multiple sensory modalities that in parallel acquire an enormous amount of information creating internal representations of the perceived world. Moreover, behaviors and skills are not innate knowledge but are assimilated by means of a knowledge acquisition process (Brooks et al., 1999) and by emotional influences (Damasio, 1994). This is also supported by the evidence that pure rational reasoning is not sufficient to realize an advantageous decision-making, as demonstrated by studies conducted on subjects with affective and emotional deficits due to brain injuries (Bechara et al., 2000).

SEAI (Social Emotional Artificial Intelligence) is a framework for the development of bio-inspired robotic control systems endowed with a form of artificial consciousness. It is specifically tailored for social robotics applications, where cognitive features aimed at giving agents the capability to perceive, process, and respond to social stimuli are mandatory. Simultaneously, it makes use of the interactions that the agent has with its interlocutors to create beliefs and internal representations that will change its behavior. In order to achieve this purpose, the system has been conceived highly adaptive, responsive but also capable of abstraction and reasoning. As in human nervous system, planning is the slower part of the control architecture. Therefore, the planning engine of the system has been implemented using a rule-based expert system, which can deal with rules and data but is not designed to be fast. In the meanwhile, sensors and actuators deal with quick reactive actions that require fast communication channels and analysis algorithms (Qureshi et al., 2004). For this reason, a hybrid deliberative/reactive architecture, which integrates a rule-based deliberative system with a procedural reactive system, has been selected as main design structure for the SEAI control system.

**Figure 2:**
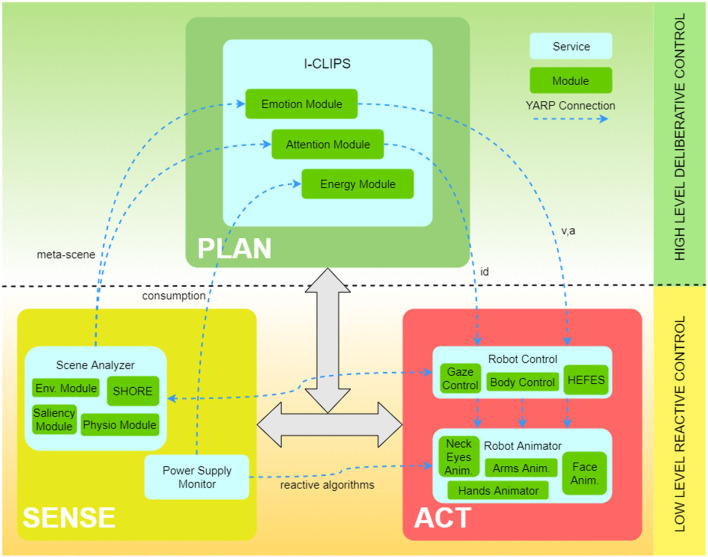
The SEAI architecture includes a set of *services* (blue boxes), standalone applications interconnected through the network. The network communication and services deploy is based on YARP, an open-source middleware designed for the development of distributed robot control systems (Metta et al., 2006). Each service has its *modules* (green boxes) that collect and process data gathered from sensors or directly from the network and send new data over the network. The information flow is defined by XML packets, a serialized form of structured data objects. Thanks to this information management, SEAI is modular and can scale up by developing services, which can even be implemented in different programming languages and placed in different hardware devices. In the proposed architecture ACT, SENSE, and PLAN blocks are only descriptive constructs. The virtual link created by the connections between ACT and SENSE services represents the reactive subsystem. Conversely, the deliberative subsystem is represented by the connections between the I-Clips Rules Engine (PLAN) service and all the other services.

As shown in Figure 2, SEAI services can be conceptually divided into three main functional blocks: **SENSE**, **PLAN**, and **ACT**.

### 4.1 SENSE

#### 4.1.1 Scene Analyzer

It is the Social Perception System (SPS) that we developed for Social Robots. This service uses dedicated modules that process incoming raw data from sensors (e.g., Microsoft Kinect ONE Camera,^1^ TouchMePad (Cominelli et al., 2017), TOI Shield^2^), extract a set of features of the social environment, and contribute to creating integrated “meta-maps,” i.e., XML files that include structured information. For example, a *meta-scene* is a structured description of the perceived social environment *(exteroception)*. The extracted features include a wide range of high-level verbal/non-verbal cues of the people presents in the environment, such as facial expressions, gestures, position, age, and gender, and a set of the visually relevant points of the scene calculated from the low-level analysis of the visual saliency map. Finally, the meta-scene is serialized and sent over the network through its corresponding YARP port. Details of the Scene Analyzer algorithms and processes are reported in Zaraki et al. (2017).

#### 4.1.2 Power Supply

It is the energy monitor of the robot. This service manages the connection with the robot power supply and monitors the current consumption and the voltage levels. The Power Supply Monitor (PSM) service calculates the robot power consumption in Watt with a frequency of 1 Hz and serializes this information to be sent over the network. Data coming from PSM constitutes part of the data used to build structured descriptions of the robot’s body state *(proprioception)*.

### 4.2 ACT

#### 4.2.1 Robot Control

This service is the first part of the robot actuation system. Its role is the translation of high-level instructions coming from the deliberative system in low-level instructions for the animators. It has internal modules dedicated to single parts of the robot (e.g., hands, arms, neck, and face). An example of these modules is *HEFES* (Hybrid Engine for Facial Expressions Synthesis), which is a module devoted to emotional control of a facial robot, described in our previous work (Mazzei et al., 2012). This module receives an ECS (Emotional Circumplex Space) point (v,a), expressed in terms of *valence* and *arousal* according to the Russel’s theory called “Circumplex Model of Affects” (Russell, 1980; Posner et al., 2005), and calculates the corresponding facial expression, i.e., a configuration of servo motors that is sent over the network to the Robot Animator. Another example is the module for the *Gaze Control* of the robot, described in details in Zaraki et al. (2014). This module receives directly from the SENSE block a meta-scene object, which contains a list of the persons, each of them identified by a unique id and associated with spatial coordinates (x,y,z). The Gaze control module is also listening to the YARP port used by the deliberative subsystem to send the subject’s id toward which the robot must focus its attention. As a result, the module sends directives to the Neck/Eyes Animator to move the gaze of the robot toward the selected subject.

#### 4.2.2 Robot Animator

It is the low-level service for the actuation of the robot. This service receives multiple requests coming from the *Robot Control*, such as facial expressions and neck movements. Since the behavior of the robot is inherently concurrent, parallel requests could generate conflicts (e.g., a surprised facial expression while blinking). Thus, the Robot Animator is deputed to the distribution of requests through each dedicated animator (e.g., hands animator, face animator, neck/eyes animator, etc.). Moreover, the animation engine is responsible for blending multiple actions taking account of the time and priority of each incoming request. This actuation service is directly connected with the motors moving the robot.

When a service of the ACT block receives an instruction coming from the PLAN block, as the example of an emotion to be expressed, then a deliberative action is taking place. On the contrary, when the instruction is a quick communication due to algorithms that link information gathered by sensors to the movement of motors, the system is dealing with a reactive non-declarative action.

### 4.3 PLAN

#### 4.3.1 I-CLIPS Brain

The name stands for *Interactive CLIPS*, it is the core of the PLAN block and embeds a rule-based expert system that works as a gateway between the reactive and the deliberative subsystems. The I-CLIPS Rules Engine has been designed using CLIPS (Giarratano and Riley, 1998), and it can be considered as the evolution of our previous work described in Mazzei et al. (2014). In CLIPS expert systems, *facts* represent pieces of information and are the fundamental unit of data used by *rules*. Each fact is recorded in the *fact-list*. I-CLIPS supports the definition of *templates*, structured facts defined as list of named fields called slots. Templates in a declarative language are structured data similar to objects in a procedural language; therefore, it is possible to convert objects in I-CLIPS templates and vice versa. The decision-making process is based on the evaluation of rules. Each rule is composed of two parts: left hand side (*LHS*) contains all the conditions to make the rule trigger, and right hand side (*RHS*) contains the actions that will be fired if the *LHS* conditions are all satisfied. The *RHS* can contain function calls, assertion of new facts or modifications of templates. Assertion of new facts generates new knowledge that can be sent to the other services through the network or used as input for the other rules. If the LHS of a rule is satisfied, that rule is not executed immediately but it is marked as *activated*. Activated rules are arranged in the *agenda*, a list of rules ranked in descending order of firing preference. Rules order in the agenda drives the execution order. Here, the I-CLIPS modules are CLIPS modules (some examples in Figure 2). Therefore, each module is a.clp file that includes definition of rules and templates. Once a module is loaded by the I-CLIPS Rules Engine, these rules and templates are defined and become part of the SEAI *Knowledge Base*. Modules are distinguished for their function. They have their own agenda and can work in parallel receiving, processing, and sending information through the network. Incoming data can be shared between more modules, as in the case of the *Emotion Module* and the *Attention Module* in Figure 2, receiving both the meta-scene, for sending different information in the network, or, no information at all, e.g., the *Energy Module*, because the outcome is a modification of internal parameters (*templates*). The modular structure of the SEAI system allows to include or exclude entire modules, and so, to unable and disable functions at run-time. Modules can have dependencies on other modules, for example, in the rules LHS of module B there can be checks about the state of templates defined by module A. If module A has not been loaded, then module B will not work, but this will not lead to any further consequences. More in general, an activation of an existing function (loading an existing module), or an addition of a new function (loading a new designed module), will not compromise the smooth functioning of the whole system.

What has been described is mainly a causal approach, similar to other approaches in the literature (Manzotti, 2006; Seth, 2008; Chella and Manzotti, 2013), but it is also possible to have partial control on time, in two ways: “prioritization” and “dummy facts.” Prioritization of the rules disposition in the agenda can be done declaring *saliency* inside the rules. Saliency is a real number from −10,000 to 10,000 that can be declared in the definition of a rule. Activated rules with higher saliency will be placed at the top of the execution list. No declaration of saliency means saliency equal to 0. With this method, layers of rules inside a module can be created. A layer, which can be considered a sub-module, is a set of rules with the same saliency that connect two or more templates, and it is called a *Rule Set*. In this way, we know that a modification of template T1 will cause a modification of template T2, and not vice versa (if not needed). If multiple rules of the same rule set are activated, they will be ordered on the agenda depending on the selected *conflict resolution strategy*. CLIPS makes available the selection of various conflict resolution strategies among which the *depth strategy* has been selected for its similarity to the typical human reasoning strategy. Using depth strategy, the last rule activated by the facts is the first to be executed generating a behavior that is more responsive and influenced by recent events. The other method is by using “dummy facts.” In this latter case, the execution order of rule sets is guaranteed by the assertion of facts: a fact (a *dummy* fact) is asserted as an action of all the RHS of the rules of the precedent rule set and as a condition in the LHS of all the rules of the subsequent rule set, which then will immediately remove that fact from the fact-list, hence the name “dummy.”

## 5 Porting the Computational Model in the SEAI Framework

With respect to the explained framework, we developed modules aimed at replicating the biological mechanisms of consciousness as described by Damasio and then formalized by Bosse. In this section, we present the developed cognitive system dividing the description into the same three notions of “emotion,” “feeling,” and “feeling of a feeling,” and we illustrate how these three levels can be exploited in SEAI for the emergence of the three-layered consciousness defined by Damasio. The “body loop” and the “as if body loop” are also discussed. Moreover, our model of the somatic marker mechanism, which was not included in the Bosse model, will be also described.

First, in order to explain how the SEAI Cognitive System processes the information, another kind of schematic representation is required. Indeed, the functioning of SEAI, akin to the human brain, resides in the structure, meaning the connections among its internal functional parts. In our case, we have a structure made of *templates* connected together by *rules*. The three level of consciousness will be described by gradually loading *modules* that will define templates and rules in the SEAI knowledge base. This schematic representation is highly inspired by the Bosse model (Figure 1), where *sensory states* are *templates* or *facts* in our system, and *local dynamic properties* are *rule sets*.

**Figure 3:**
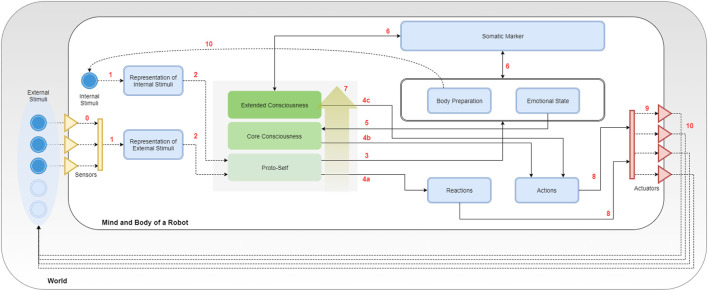
Porting Bosse in SEAI. Key numbers are used for description in section 5.3.

In Figure 3, the entire SEAI Cognitive System is shown, where all the developed modules have been loaded.

### 5.1 The External World

In Figure 3, the line delimiting the big white box represents the edge of the physical body of the robot, the gray box in which it is immersed is the external world. Sensors and actuators are the interfaces by which the robot connects with the world. They are represented by a collection of triangles standing in the middle between the body of the robot and the world. Incoming yellow triangles are sensors and outgoing red triangles are actuators. The set of sensors and the perception capabilities depend on the features and the equipment of the robot. As represented in the figure, there are external stimuli that can be perceived by the perception system (bright blue circles), while others (pale blue circles) may not have the corresponding sensory channel in the perception system of the robot. In the case of social robotics, stimuli could be different features of the environment (e.g., temperature, noise level, luminosity, and so on), social cues regarding a unique subject (e.g., gender, facial expression, posture, physio parameters, and so on) or characteristics of an object (e.g., shape, color, dimensions, and so on). Usually, each sensor has a dedicated perception module for the pre-processing of extracted raw data. This is similar to the pre-processing taking place in the human sensory channels. Likewise, the actuation system depends on the motor system of the artificial agent. Typical actuators are servomotors and a set of motors corresponds to a body part of the robot driven by a dedicated animator. However, also speakers for speech synthesis or lights simulating blushing of the skin are considered here as actuators. Arrows coming out from actuators represent the actions of the robot that will lead to some change in the world, this change will be reacquired by the agent as a new collection of external stimuli.

### 5.2 The Internal World

In the model of Figure 3, the focus is all on the PLAN block, which has been extended and its internal structure revealed. The SENSE and ACT block have been compacted in two representational bars with the same reference colors used in Figure 2: the yellow bar represents the sum of all perception services, while the red bar stands for the actuation services. Blue boxes are *templates*, and continuous arrows are *rule sets*. Directions of arrows represent the causal/temporal direction due to the abovementioned layering approach. In parallel with external stimuli, the agent has also internal stimuli. They are represented in the schema as an inner blue circle and can be a collection of simulated physiological parameters or a set of values representing the psycho-physical state of the agent. Internal stimuli are updated after every execution cycle after processing the information coming from the external and internal world of the agent. In the middle of the picture, it can be noticed a gray square containing three representative layers. The gray space is the working memory of the robot and corresponds to the “fact-list,” the list of all the facts of which the agent is aware of itself and the world. The three representative layers are a symbolic representation through which we describe the arise of consciousness that is reached and enriched by the awareness of facts of increasingly higher level of abstraction. Non-continuous arrows are not rule sets but YARP connections with other services or another kind of connections. These details will be clearer with the following description of rule sets and modules.

### 5.3 Rule Sets and Modules

Following the key numbers in Figure 3: **(0)** external stimuli reach the SENSE block passing through sensors; these connections indicate the sensory acquisition, pre-processing, and integration. These two latter processes take place in the SENSE and provide a single structured meta-map (e.g., a meta-scene) that is sent through a YARP connection. Once the information has been extracted by the external world (exteroception) or perceived from the body (interoception) forming meta-maps, these are analyzed by the deliberative system. **(1)** The system uses pattern matching to compare incoming information with internal representations (pre-defined templates) and recognize real and useful information from inconsistent and useless data. **(2)** If a meta-map has an expected structure and satisfies conditions about internal data, then it is accepted by SEAI as reliable information, and a new fact is asserted in the agent working memory. Facts in the fact-list activate sets of rules of the I-CLIPS rules engine, which will modify other templates or create secondary facts. **(3) EMORS** (EMOtion Rule Set) is a set of rules that analyze facts to process a related emotional predisposition, realized as a modification of values of the templates *body preparation* (bp(v,a)), *emotional state* (es(v,a)), or both. **(4) BEHRS** (Behavioral Rule Set) is the set of rules that analyze the facts to provide instructions for the robot about certain actions to take, the effect of these rules is the modification of the templates *reactions* or *actions*. This rule set is divided into **(4a) STD-BEHRS** (STandarD Behavioral Rule Set), **(4b) ALT-BEHRS** (ALTernative Behavioral Rule Set), and **(4c) SPEC-BEHRS** (SPECific Behaviors Rule Set), which have increasing priority. This distinction will be clearer in the next section. **(5) FEERS** (FEEling Rule Set) analyze the emotional state template to extract a higher level information that is a conscious feeling, the consequence is the assertion of a secondary fact about the mood of the agent. **(6) SOMARS** (SOmatic MArker Rule Set) is the set of rules simulating the somatic marker mechanism. These rules work in two different directions: they can analyze the body and emotional state to trigger the assertion of a somatic marker, and in case of recognition of a marked entity, they can recall the bodily state that the agent “felt” when that entity was labeled. **(7) REARS** (REAsoning Rule Set) is the set of rules that allows reasoning chain and deductive inferences. These rules do not connect specific templates, because they analyze known facts to assert higher level facts. This rule set is extremely useful to do abstract symbolic reasoning and contributes to the modeling of higher levels of consciousness. Thereby, it is represented by a golden arrow inside the fact-list box. **(8) EXERS** (EXEcution Rule Set) must be the last set of rules to be run. Therefore, they have the lowest saliency values and will be placed at the bottom of the agenda. When all the other rule sets have contributed to the modification of the templates, the actions to take have been decided, the EXERS can send instructions to the ACT Block. This is done through function calls in their RHS that send high-level commands in the YARP network. **(9)** These commands are translated by the Robot Control into motor commands and dispatched by Robot Animator to the actuators of the robot. **(10)** Finally, the bodily state induced by the events is upgraded as a new set of internal stimuli, and the actions of the agent lead to a modification of the social environment that is interpreted as a new set of external stimuli. An execution cycle from 0 to 10 lasts 0.33 ms, which is in line with the physiological time needed for passing from an intention to an action (Libet et al., 1983).

The discussed rule sets and templates are arranged in three different modules:

**EMOTION MODULE** includes the following: *Representation of Internal Stimuli* template, *Representation of External Stimuli* template, *Reactions* template and *Body Preparation* template. As Rule Sets, the Emotion Module includes EMORS, STD-BEHRS, and a few rules from REARS and EXERS;
**FEELING MODULE** includes the following: *Emotional State* template, *Actions* template, additional EMORS rules that can modify also (or only) the emotional state, ALT-BEHRS, an extension of REARS, and additional EXERS rules for the execution of actions;
**FOF**^3^ **MODULE** includes the following: *Somatic Marker* template, SOMARS, SPEC-BEHRS, and additional rules of REARS.

As can be noticed, there are entire rule sets that are sole property of a module (e.g., SOMARS) and rules of the same rule set that appear in different modules (e.g., EMORS and REARS). In fact, different modules may include rules with similar function, connecting the same templates, or having the same priority.

### 5.4 Emotion and Proto-Self

**Figure 4:**
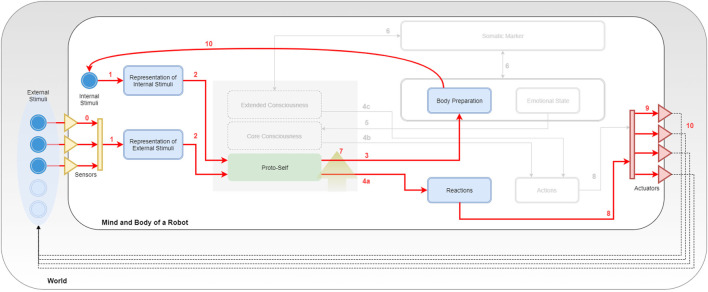
SEAI with only the *Emotion Module* loaded.

Following the narrative process used in Bosse et al. (2008), we start from a SEAI system in which only the *Emotion module* is loaded (Figure 4). Included in the Emotion module, there is the *body preparation* template. As mentioned in the description of the SEAI framework, to model emotion we use the ECS (Emotional Circumplex Space) representation (Russell, 1980). An ECS point is described by two coordinates: *v, valence*, the quality of an emotion (i.e., positive or negative), and *a, arousal*, which is the activation level of an emotion; *v* and *a* are normalized between 1 and −1. Body preparation is described by a (*v*,*a*) point that is a bodily state, induced by events, that corresponds to a specific emotion. This state will be performed by the agent as an immediate reflex and will last only the duration of the emotional stimulus. Let us assume the same example reported in Bosse et al. (2008), an agent hearing and reacting emotionally to music, and suppose that the SENSE block of SEAI includes a simple software for sound analysis. For example, this software is able to extract the music tempo in terms of beats per minute (bpm) and the sound volume (db). Then, referring to Figure 4, this example in SEAI would be the following: **(0)** the music (external stimuli) is acquired by the sensors of the agent (microphones), the audio is processed by the application in the SENSE block, which creates a single structured data: a meta-map containing the perceived characteristic of that music. The meta-map is sent as a YARP bottle in the network; **(1)** the meta-map comes to the I-CLIPS Brain, where is compared with the representation of music, a template (music (bpm) (volume)); **(2)** if the information is consistent (e.g., a condition could be *bpm* > 0) then the meta-map becomes a fact in the fact-list, otherwise is rejected; **(7)** REARS may be activated by the (music) to do reasoning chain and assert facts, such as (music-genre-is chill-out) if 70 < *bpm* < 120 or (volume-is low) if *db* < 45; **(3)** the appearance of a (music) fact activates also the EMORS. For instance, EMORS can trigger specific bodily states in relationship to specific volume ranges. This means a modification of *body preparation* from neutral bp(0,0) to bp(v,a); **(10,1,2)** this bodily change is updated as an internal stimulus and becomes also a fact in the fact-list; **(4a)** the contemporary presence of the two facts, one about the music and one about the bodily change, activates a behavior, typically a rule of BEHRS which acts on the *reactions* template, copying the bp (*v*,*a*) coordinates that now are present as a fact of the fact-list; **(8)** when a disposition is ready and available in the *reaction* template, EXERS is activated and the (*v*,*a*) point is sent to the ACT block; services of the ACT block interpret and express the emotional state to perform, translating that emotion in a list of commands for motors. In this way, the emotion is physically expressed through the body of the agent (e.g., a serene facial expression).

This part of the process corresponds to the sequence **LP0**, **LP1**, **LP2**, and **LP3** described in section 3. At this stage, the system is only responsive and capable to process information and express consistent emotional states. The behavior of the agent will be always the same in front of the same stimulus, and its reactions will not last more than the duration of the incoming input. In any case, the simultaneous existence of known facts about the surrounding environment and the body state induced by the entities of that environment fully satisfy the definition of Proto-Self. As a consequence, this first preliminary stage of synthetic consciousness results activated in Figure 4.

### 5.5 Feelings and Core Consciousness

**Figure 5:**
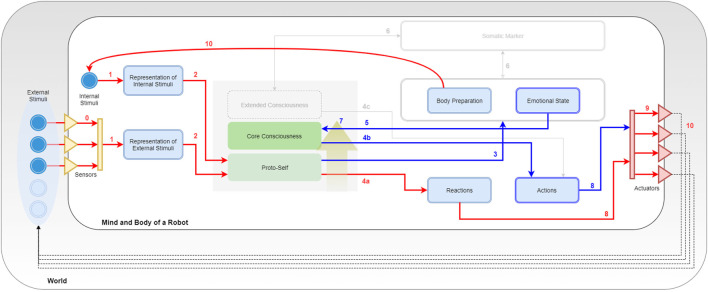
SEAI after *Feeling Module* loading. New parts highlighted in blue.

The addition of the *Feeling Module* leads to the definition of new templates and rule sets, which have been highlighted in blue, in Figure 5. A new template defined by this module is the *emotional state* template. This new internal representation of the cognitive system is different from *body preparation*. On the one hand, the same emotion model is used for the representation, and so, the instances of this template are also ECS points. On the other hand, es(v,a), unlike bp(v,a), is an internal parameter that does not lead necessarily to an immediate reaction, but rather it is used by the system to modulate the behavior of the robot. This modulation occurs because the module defines new rules of EMORS, which can modify bp(v,a), es(v,a), or both. The bp(v,a) points are still discrete states, while es(v,a) is modified gradually, by an increase or decrease of its previous (*v*,*a*) values. The FEERS checks *emotional state* to assert in the fact-list the current emotional state as a fact. REARS will interpret these states to assert secondary-order facts about the current mood of the agent (e.g., bored, relaxed, and annoyed). The simultaneous presence in the fact-list of a bp to perform and an es will activate the ALT-BEHRS, which acts on the *actions* template, placing (*v*′,*a*′) values that correspond to

$$\begin{array}{l} v^{\prime }=(k-1)*v_{bp}+k*v_{es}\\ a^{\prime }=(k-1)*a_{bp}+k*a_{es}, \end{array}$$

where *k* is the *influence factor*, a global variable, accessible to all modules, which value is set within 0 < *k* < 1 and determines the influence of the emotional state on the agent.

Returning to the example of music listening, nothing changes until the sensory representation of the music is asserted as a fact in the fact-list, but now **(3)** new EMORS rules determine variations of the es values. For example, there is a rule that makes *v*_es_ increase together with the music tempo and another one making *a*_es_ decrease in case of low sound volume. Let us take the case of a slow relaxing music heard at low volume. A protracted listening to this kind of music will lead to: **(5)** the assertion of the fact es(v,a) by the FEERS, which every run cycle will be upgraded with decreasing values of both *v*_es_ and *a*_es_; **(4b)** the activation of the ALT-BEHRS due to the contemporary presence of a bp and an es in the agent working memory; **(7)** the analysis of the es-fact by the REARS and the subsequent assertion of secondary-order facts (e.g., (music-is boring)). The ALT-BEHRS acts on the *actions* template placing (*v*′,*a*′) values. **(8)** The EXERS rules defined by the *Feeling module* have higher saliency than the EXERS rules of the *Emotion module* and check the *actions* template. When all the BEHRS rules have been fired, if both *actions* and *reactions* are filled with values, reactive impulses are temporarily “inhibited” and actions are sent to the ACT block services. The follow-up **(9,10)** is exactly the same described in the previous condition because services of the ACT block are not aware of the declarative process underlying the received instruction. Nonetheless, thanks to *Feeling module*, we will see the previous serene facial expression turning gradually into a bored expression.

The described process corresponds to the addition of **LP4** and **LP5** in the computational model and the emergence from the subcortical to the cortical level in the biological model. It represents the arise of a feeling through the *body loop*. Indeed, the result of this cognitive process is the emergence of secondary-order representations generated by means of slower gradual changes in the body. Here, feelings are not yet internally represented. At this stage, the agent has not a specific behavior toward a precise evocative object, thus, cannot even speak about the music. Nonetheless, reactions to the music are changing, the raised emotions are changing, and feelings are getting clear, which corresponds to the description of what Damasio calls a *Core Consciousness*, that appears activated in Figure 5.

### 5.6 Feeling of a Feeling and Extended Consciousness

In order to uplift feelings and consciousness to a higher level, we relied on the somatic marker hypothesis, formulated by Damasio (1994). A *Somatic marker* (SM) is an association between a relevant change in the body state, perceived as an emotion, and the causative entity that induced that change. According to the hypothesis, somatic markers are processed in the ventromedial prefrontal cortex (VMPFC) and the amygdala and strongly influence subsequent decision-making. Indeed, SMs use our body to create emotional beliefs and opinions about specific entities with which we interact, giving an essential contribute for the formation of an extended consciousness. This mechanism, in case of a second exposure to a marked entity, will recall the body state felt in the past biasing our decisions and behavior toward that specific entity. The hypothesis was demonstrated by Bechara et al. submitting healthy patients and brain-injured patients to the “Iowa Gambling Task,” a gambling card game specifically conceived by the authors to assess the efficiency of the SM mechanism (Bechara et al., 1997). To model this brain–body mechanism, we designed the SOMARS. This part of our cognitive system has been tested in a preliminary computational experiment, where we submitted a simulated reproduction of the Iowa gambling task to an artificial agent endowed with SOMARS (Cominelli et al., 2015).

**Figure 6:**
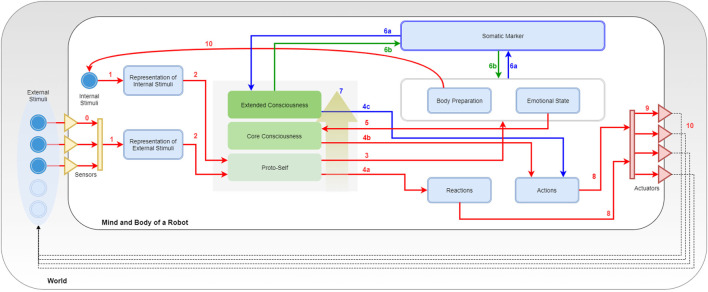
SEAI after *FOF Module* loading. New parts highlighted in blue. Green arrows (6b) indicate SOMARS rules for somatic marker recall.

In Figure 6, the SEAI system after the loading of the FOF module is shown. This leads to the definition of the *Somatic Marker* template, additional rules in REARS, the SPEC-BEHRS, and SOMARS. SOMARS has been divided into SOMARS rules for SM creation (6a, blue arrows in Figure 6) and for SM recall (6b, green arrows in Figure 6). To better explain the labeling and recall method, we refer again to the music example: nothing changes in the perception of the music **(0,1)** and the creation of its internal representation as a fact **(1)**; neither the influence of the music on body preparation and emotional state through the EMORS is changed **(3)**, nor the subsequent feelings assertion due to the FEERS **(5)**; but now there are rules of SOMARS that, **(6a)** if the intensity of the emotional state |*es*|, intended as the modulus of *es*(*v*,*a*) vector, exceeds a decided threshold called *sensitivity* (*s*), then assert a fact in the fact-list: an instance of the *somatic marker* template. A somatic marker in SEAI is a fact (sm(id)(value)(bp)), where *id* is an identification number assigned to the causative entity, *value* = *v_es_* * 100, and bp is a multifield slot that contains the current (*v_bp_, a*_bp_). In the example, the listened music, after a few minutes playing, induces by means of EMORS an es, which modulus is

$$|es|=\sqrt{v_{es}^{2}+a_{es}^{2}}>s,$$

as a consequence, SOMARS checks the fact-list, the music-genre chill-out is identified with a specific id, labeled with a value and associated with the bp(v,a) felt in that moment. A new (sm) has been created.

This sequence corresponds to the sequence of transitions between the states *s0* (*the proto-self exists at the inaugural instant*), *s1* (*an object come into sensory representation*), and *s2* (*the proto-self has become modified by the object*). In LEADSTO formalization, this is equivalent to **LP7**, **LP8**, and **LP9**.

From here on, the labeled entity in the fact-list will activate rules of the SOMARS for SM recall **(6b)** that will modify the body preparation state immediately recalling the bp(v,a) that was felt and associated with that entity. This bp will be represented as a sensory representation of the body state (sr(S) in Bosse, a fact in SEAI). This new state is not derived by an upgrade of the body state (**LP4** in Bosse, **10** in SEAI), but from an internal representation of body preparation recalled from the long-term memory of the agent. This is, in all respects, an *as if body loop*, and corresponds in LEADSTO notation to **LP6**.

Another consequence of the recognition of a marked entity may be the activation of **(4c)** a rule of SPEC-BEHRS, triggering some specific behavior toward that entity, pushing a high priority action to be executed, such as saying something about that music (e.g., *“this music is getting boring”*). The sequence that includes **(4c)**, **(8)**, and **(9)** coincides to **LP10**.

Finally, even REARS rules may be activated to assert more abstract and general facts. For instance, a rule of the reasoning rule set could be: if there are the facts (music), (music-genre is chill-out), and a (sm) which label that music with a bp corresponding to a bored face, then assert the fact (chill-out is boring).

The emergence of SMs is the emergence of personal opinions, about the entities of the world, that the agent autonomously builds through the interactions with such entities. This mechanism, which leads to the construction of an autobiographical memory and biases the behavior of the agent and its opinion about the world, is deputed to the bio-inspired mechanism activated by the FOF module. Things would have ended differently, for example, if other entities of the external world had moved the emotional state in a different direction, predisposing the agent in a better “mood.” In this case, chill-out music would have been probably labeled as a nice music genre recalling a pleasant body state to express. In general, it is evident that this level of consciousness, which could not exists without its predecessors, moves beyond the “here and now,” includes personal opinions and feelings about specific entities of the world and allows the creation of higher general thoughts. We identify this level with the equivalent of the *Extended Consciousness*, which as a consequence appears activated in Figure 6.

## 6 Testing SEAI in the Real World—The HRI Experiment

**Figure 7:**
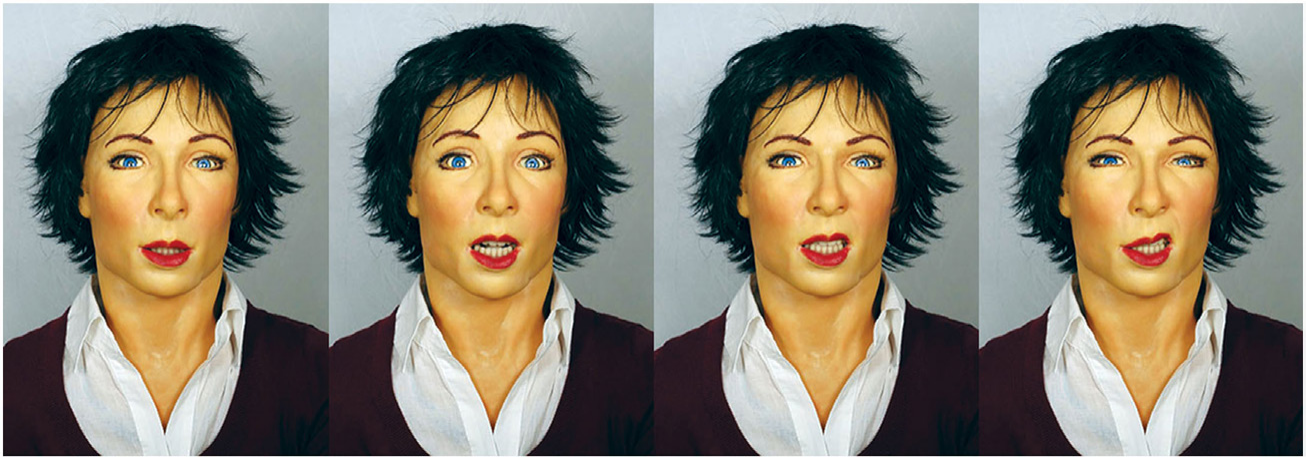
The FACE Robot (Facial Automaton for Conveying Emotions) displaying some of its hyper-realistic facial expressions.

In this section, we report an experiment in which SEAI has been used as cognitive system of the humanoid robot FACE (Facial Automaton for Conveying Emotions)^4^ (Figure 7). FACE is a human-like robotic head, with the appearance of an adult female, capable to perform very sophisticated expressions by means of a hyper-realistic facial mask. The android’s head has been customized by our research team starting from a Hanson Robotics^5^ head. The facial mask is made of Frubber (“flesh rubber”), a proprietary skin that mimics real human musculature and skin, and it is actuated by 32 servomotors. The robot has also a mechanical system, composed of a controlled neck with 3° of freedom and movable eyes to allow gaze control (Zaraki et al., 2014, 2017). In this experimental setup, the head has been mounted on a passive mannequin, placed in a seated position. In order to achieve the maximum possible naturalness of the HRI, the interaction takes place in a normal situation of everyday workplace: an office room that has not been prepared or specifically structured. The experiment of this study has been approved by the Ethics Committee of the University of Pisa (prot. 68459, ref. Ethical Approval by CEAVNO, Comitato Etico di Area Vasta Nord). All research participants provided written and informed consent.

In the presented experiment, FACE interacted with three subjects, identified as ID1, ID2, and ID3. The experiment can be divided into the following four scenes:

**Scene 1**. ID1 enters the room where the robot is seated. He performs several disturbing or impolite actions: he does not greet the robot, immediately invades the robot’s intimate space, does not speak to it, folds his arms for a while, and then leaves.
**Scene 2**. ID2 enters the room and performs mixed actions: he greets robot, invades the robot’s intimate space but then immediately makes a step back, speaks for a while to the robot, and then leaves.
**Scene 3**. ID3 enters the room and performs actions that are typical of nice behavior: he greets warmly the robot, smiles at it, speaks a lot to it; finally, greets again and leaves.
**Scene 4**. ID1, ID2, and ID3 come back into the room where the robot is located and arrange themselves in three positions at different distance from the robot. They just maintain their position for about 30 s without doing anything to draw the attention of the robot. Then, they all leave the scene.

This sequence has been recorded as a repeatable scenario using Kinect Studio, a tool to record and play back depth, color streams, and audio from a Kinect.^6^ In this way, it is possible to present exactly the same scenario to the robot comparing the effect of the same social scene in three different conditions of the cognitive system: (**cond1**) SEAI with only the Emotion module and the *Attention module*; (**cond2**) including the *Feeling module*; and (**cond3**) including the *FOF module*.

Images gathered by the Kinect are analyzed by the Scene Analyzer, which extracts (or estimate) several main social cues of the subjects involved in the scene, e.g., their facial expression, age, gender, gestures, body postures, and proximity. The SENSE service detects also, for every incoming frame, the *salient point* of the image, processed by means of pure image analysis based on colors, contours, light contrast, rapid movements, etc. This point is also identified by an ID, which is ID0. All the information is organized as a *meta-scene* that is sent to the I-CLIPS Brain through YARP. Once the meta-scene has been processed by the I-CLIPS Brain, an ID will draw the attention of the robot that will look at it. This ID is also called *Winner ID*. This is an automatic non-emotional mechanism decided by the rules of the *Attention module*, loaded in all the three conditions. This module, indeed, defines several standard behavioral rules (STD-BEHRS) that, choosing the winner, drive the attention of the robot. For example, the FACE attention is attracted by someone raising their hand or speaking to the robot. If no one is doing anything relevant but subjects are present in the scene, then the robot will look to the closest subject. If no subject is present in the FOV, then the robot will analyze the scene by looking at the salient point. The attention model, here implemented in the form of rules, was studied and discussed in Zaraki et al. (2017).

### 6.1 Results

#### 6.1.1 Experiment 1

**Figure 8:**
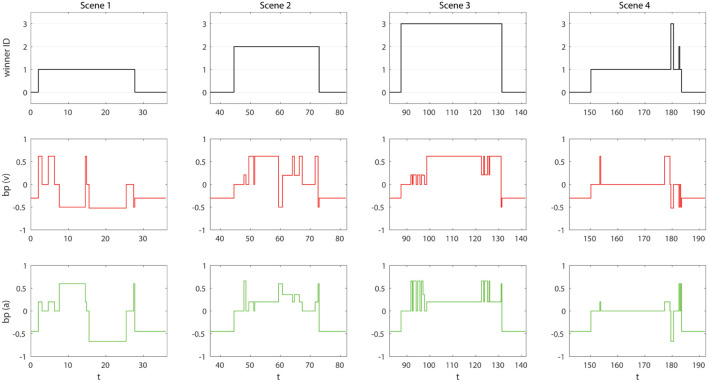
Results of the HRI Experiment with FACE integrating SEAI in condition 1. Columns are the four scenes. Rows are, in order: *winner ID, bp (v)*, and *bp (a)*. Time *t* is expressed in seconds.

In this first condition, the *Emotion module* is loaded. This leads to the definition of *body preparation* and the EMORS that can modify bp (*v, a*) according to external and internal stimuli. It results in a FACE bodily change, and so, an emotional response to what is happening in its social environment. For example, the absence of people in the FOV of FACE causes the display of a sad facial expression corresponding to negative valence and low arousal (−0.3, −0.5). As the subject enters in the room, we see in Figure 8 two parallel consequences: rules of the *Attention module* will bias the attention of the robot from the salient point to the detected subject, while rules of the *Emotion module* change the bodily state of the robot. This change in the status of the body will be expressed according to our emotion model through the FACE expressive capabilities: an ECS point is translated by the Robot Control in 32 commands for the relative servomotors moving its face and neck.

In Figure 8, looking at the charts of bp(v) and bp(a), it is possible to see, along all the interaction, the emotional response of the robot. FACE expresses discomfort (−0.5, −0.6) when a subject invades its intimate space, an angry expression (−0.52, −0.67) if someone folds his arms, smiles (0.21, 0.6) if someone greets her or smiles at her, and expresses interest (0.62, 0.2) when an interlocutor speaks to her. Without going into the details of the actions performed by the subjects in their interaction with the robot, the trend of bp (*v*,*a*) shows how the robot is emotionally affected in the three first scenes. In the first one, the impolite behavior of ID1 induces unpleasantness and annoyance, hence, values of negative valence are predominant, accompanied by large arousal fluctuations. ID2 has an engaging interaction with the robot, he manifests a polite behavior, quite neutral. As a consequence, positive values of valence are predominant and the arousal is not highly affected. In scene 3, we can see the effects of the interaction with ID3: the interaction is full of positive stimulus, this induce in the robot frequent emotions of pleasantness and high excitement. Finally, we see in scene 4 that, the entire time the robot is detecting people, bodily changes are nearly irrelevant. Indeed, the three subjects just stand in front of the robot without saying or doing anything. The emotion expressed by the robot is always neutral (0,0), with an exception when the subjects leave the room. In this transition, there are fluctuations due to the overlapping of detected people going out through the same door, resulting in a difficult reconstruction of the skeletons by the Scene Analyzer. In any case, sudden quick variations are filtered by the Robot Animator and will not lead to the movement of the robot.

Concerning the behavior of the robot, in terms of attentive model, for the first three scenes, the winners of FACE’s attention can only be the single subject presents in each scene or the salient point (ID0). The salient point draws the attention of the robot in the absence of social stimuli, therefore, before and after subjects’ detection. In the last scene, including all subjects, the robot focuses its attention on ID1, because he is the closest subject and nobody is doing anything to draw the attention of the robot.

At this stage, FACE bodily state is clearly affected by external events, but the agent is not aware of its own feelings. Emotions last exactly the duration of the stimuli. There is no memory of the experiences. Therefore, behavior is reactive and FACE does not take deliberative decisions about specific subjects. The evidence is that when the subjects come back into the room it is like nothing has happened before, the attention of the robot is not influenced and the robot simply look at the nearest person. We are still at an equivalent of the proto-self level of consciousness.

#### 6.1.2 Experiment 2

**Figure 9:**
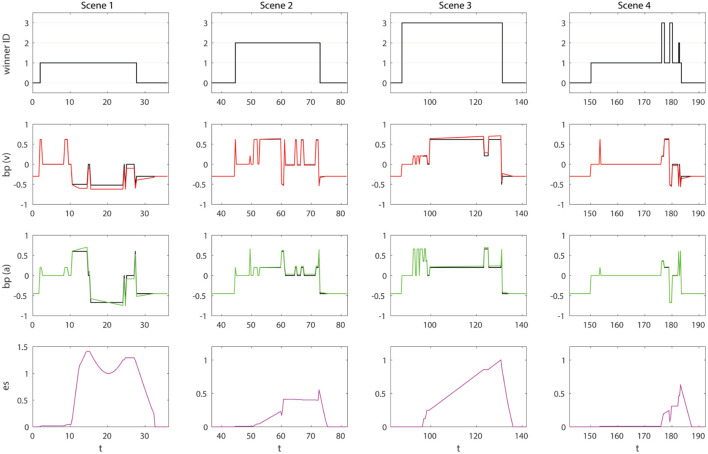
Results of the HRI experiment with FACE integrating SEAI in condition 2. Columns are the four scenes. Rows are in order: *winner ID, bp (v), bp (a)*, and *es*. Effectively executed *v*′ and *a*′ are, respectively, colored as red and green lines, while *bp* values not affected by *es* are represented as black lines to allow comparison. Time *t* is expressed in seconds.

The emotional state template comes along with the loading of the *Feeling module*. The effects of this module are shown in Figure 9. EMORS can now modulate the emotional state (es) of the agent, which is continuously upgraded by FEERS through the assertion of facts in the working memory. The influence of events on *es* can vary from a low influence (e.g., talking to the robot, as in scene 3, from *t* = 100 s to *t* = 130 s) to a very important influence (e.g., invading its intimate space, as in scene 1, from *t* = 10 s to *t* = 15 s). This leads to a modification of the emotional state expressed by the robot: the agent does not show exactly the (*v_bp_, a_bp_*) values, but this emotional immediate reaction is modulated by the new internal representation of emotions. These new values are (*v*′,*a*′), discussed in section 5, where the *influence factor* has been set as *k* = 0.1. The higher priority of ALT-BEHRS guarantees that (*v*′,*a*′) are executed instead of (*v*_bp_, *a*_bp_). In the charts of bp(v) and bp(a), we report both the values with (red line for valence, green for arousal) and without (the black line underneath) the es contribute. As expected, their difference is proportional to the intensity and the duration of the emotional state perceived. Moreover, the trend of es is slower and can last more than the duration of the causative stimulus, as in the transitions from detecting subjects to loneliness, which is no more immediate but smoothed (e.g., scene 3, es and bp after *t* = 130).

At this stage, the agent is aware of its own simulated feelings thanks to a continuous assertion of facts in its working memory reporting its own synthetic emotional state. Feelings also emerge in the body as shades of the emotional states expressed by the agent. In any case, all this information is temporary, there is a modulation of the behavior but still, no clear connection between the causative stimulus, the agent body state, and the subsequent feeling perceived. As a consequence, a recall of emotions driving specific behaviors is not feasible and the deliberative behavior of the agent is approximately the same: ID1 is still the winner of FACE attention.

#### 6.1.3 Experiment 3

**Figure 10:**
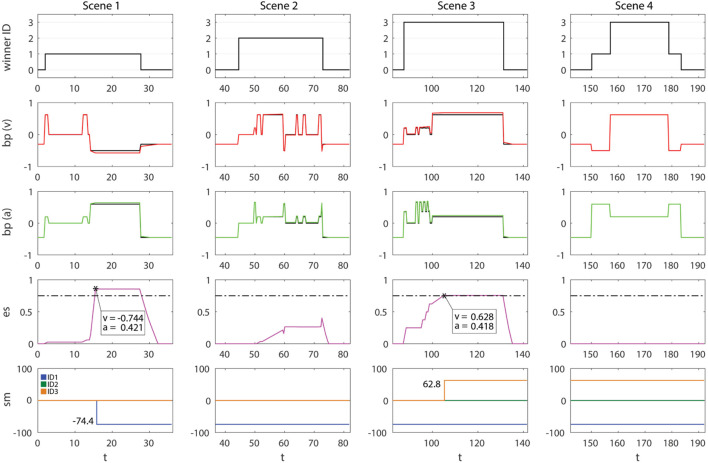
Results of the HRI experiment with FACE integrating SEAI in condition 3. Columns are the four scenes. Rows are, in order: *winner ID, bp (v), bp (a), es*, and *sm*. Effectively executed *v*′ and *a*′ are, respectively, colored as red and green lines, while *bp* values not affected by *es* are represented as black lines to allow comparison. In *es*, we pointed out the *es (v,a)* values that caused the creation of a *sm*. In *sm* we reported the *values* of SMs in the moment in which they have been created by the system. IDs colors are indicated in the *sm* chart. Time *t* is expressed in seconds.

The addition of *FOF module* results in the definition of SOMARS and the possibility for SEAI to exploit the somatic marker mechanism. In Figure 10, we can see the results of the experiment in this third condition. The difference is impressive: during the first three scenes, in which the agent interacts individually with the three subjects, the attentive behavior of the robot is exactly the same, but the emotions evolve in a very different way; while, in scene 4, in front of all the subjects the attentive behavior is completely changed, emotional reactions are more stable, and the emotional state perceived is zero. This is due to the SM creation and recall mechanism discussed in section 5.6. Referring to the experiment, sensibility has been set to *s* = 0.75, so, the annoying behavior of ID1 makes the es intensity increase rapidly until it exceeds the *s* threshold (*t* = 15.5 s), this leads, in the next run cycle (*t* = 15.83 s), to the creation of a SM containing the *winner ID*, a marker *value* of −74.4 according to the equation reported in section 5.6, and the current *bp (v,a)* induced by the causative entity. The same thing is happening when FACE interacts with ID3 during scene 3, but here the quality of the marker is positive (details in Figure 10). As soon as these markers are created, the emotional state is no longer perturbed by the marked entity, because the agent has a precise belief and an associated emotional behavior to express toward that specific subject, which is the somatic state felt and labeled through the somatic marker mechanism. This can be seen both in scenes 1 and 3 after the creation of the SM, and, which is more important, in the last scene. Indeed, in scene 4 when all the subjects are in front of the FACE robot, FACE is no longer attracted by the presence of the nearest subject. On the contrary, the presence of marked subjects completely bias its behavior: ID1 now is labeled, and when he enters and becomes detected, the robot immediately recalls the somatic state (−0.5, 0.6) felt in the past causative interaction; the same happens as soon as ID3 comes into the FOV of the agent. In our behavioral model, SPEC-BEHRS related to positive marked entities have higher priorities on rules driving the attention on negative marked entities. Therefore, until ID1, ID2, and ID3 are all detected, the attention of the robot is all for ID3. FACE is specifically attracted by him, thanks to his previous nice behavior, and stares at him with a pleasant facial expression (0.2, 0.68). In this last scene, ID2 becomes quite invisible to the robot, because his neutral previous interaction has never pushed the emotional state over the sensibility threshold (as shown in the es trend of scene 3). That experience did not influence enough the robot to create a dedicated SM.

This last experiment represents the test of the full SEAI system configured as Damasio’s theory simulator endowed with the somatic marker mechanism. At this stage, the agent is able to autonomously create long-term memory information about entities of it social environment. These memories are emotional memories and are perceived by means of the body. They can affect the somatic state of the agent in case of further interactions, and bias the behavior in a very evident way. This mechanism, completely bio-inspired, let the agent automatically build its own beliefs about the outer world and about itself. What has been described, to all intents and purposes, models the construction of an autobiographical emotional memory and it respects the minimum requirements for the emergence of what Damasio described as an *Extended Consciousness*.

## 7 Discussion and Conclusions

In this paper, a novel cognitive architecture for social robots has been presented. We selected a well-known mind theory to be modeled and implemented in the form of a cognitive system controlling an emotional robot with sophisticated expressive capabilities. The developed system is called SEAI (Social Emotional Artificial Intelligence). In particular, it has been inspired by the findings of Antonio Damasio and it is consistent with the computational formalization made by Bosse et al. (2008). It is based on a declarative rule-based expert system on top of procedural services deputed to the perception and motion control of the robot. Compared to other robotic cognitive systems, some of which discussed in the state-of-the-art section, SEAI has still some shortages: homeostasis control is missing, the agent’s physiological parameters are a symbolic representation, capabilities such as perspective-taking or mind-reading have been not yet considered. Most of the effort has been spent in the C1 meaning of consciousness, rather than in the C2 definition (Dehaene et al., 2017). On the other hand, SEAI stands out from the other systems thanks to the hybrid concept with which has been designed. Indeed, the modular design of the architecture potentially enables the extension and portability of the system to any other social robot simply adapting, or adding, low-level services to the sensory apparatus and the motor system of the specific agent. This can be done keeping the “personality,” memories, beliefs, experience, and behavioral traits of the agent, all of which depend on the cognitive part of the system, and therefore can be transferred or modified independently. Moreover, the innate extensibility of the rule-based expert system, which is the core of the cognitive block, puts no specific limitations to the inference reasoning capabilities with which the artificial agent can be endowed, which depends on the number and complexity of the rules. In the presented experiments, SEAI endowed a social humanoid with artificial emotions and feelings that have been influenced by the context, the agent managed to exploit them to build opinions on the social world in which is immersed, and, based on them, it manifested more sophisticated social skills. For instance, in the last experiment, an evident bias from the robot’s standard behavior emerged. Such experiment obviously does not pretend to be the demonstration that we created a conscious being, but it is a clear demonstration of how SEAI and the chosen “understanding by building” approach lead to an important confirmation: with SEAI, robots can benefit from their own artificial emotions for taking decisions and treasure their past interactions. Future works will include (1) the expansion of SEAI in order to include the missing features identified in the other robotic cognitive systems; (2) the simulation of many other complex human social behaviors by writing new rules and expanding the current rule- sets; (3) study of the people’s reactions to the adaptation of the robot behavior to its social environment by means of HRI experiments, eventually on long-term interactions. For the purpose of points (2) and (3), the involvement of professional figures from behavioral psychology and neuroscience would be greatly fruitful, and a questionnaire investigating the interlocutors feedback about the perceived consciousness of the robot will be required. The key issue is if the social interaction with humans would effectively benefit from the created deviations in the behavior of the social robot. Our hypothesis to test is that the realism derived by the integration of SEAI will improve the acceptability and the believability of this new kind of robots. In conclusion, we believe that SEAI is a potential valuable tool for modeling human consciousness and, ultimately, a promising beginning to tackle the possibility to attribute to the robots a synthetic form of consciousness. In this latter case, ethical issues will become extremely relevant and critical.

## Ethics Statement

The experiment of this study has been approved by the Ethics Committee of the University of Pisa (prot. 68459, ref. Ethical Approval by CEAVNO, Comitato Etico di Area Vasta Nord). All research participants provided written and informed consent.

## Author Contributions

LC is the main author of this paper, he formalized the Damasio’s theory in order to port it on the SEAI system, wrote the rules of the expert system, and designed the experiments to test the SEAI efficacy; DM designed the software architecture at the core of the SEAI framework; DR is full professor of bio-engineering and robotics who drove the focus of the entire research proposing Damasio as consciousness model to be analyzed. DR also did a strong contribution to the organization, writing, and proofing of the presented paper.

## Conflict of Interest Statement

The authors declare that the research was conducted in the absence of any commercial or financial relationships that could be construed as a potential conflict of interest.

## Footnotes

^1^https://developer.microsoft.com/en-us/windows/kinect/hardware.

^2^http://www.besos.cc/portfolio-articoli/toi-shield/.

^3^FOF, Feelings Of Feelings.

^4^www.faceteam.it.

^5^http://www.hansonrobotics.com/.

^6^https://msdn.microsoft.com/en-us/library/hh855389.aspx.
